# Multi-Locus Sequence Typing of Enteroaggregative *Escherichia coli* Isolates from Nigerian Children Uncovers Multiple Lineages

**DOI:** 10.1371/journal.pone.0014093

**Published:** 2010-11-23

**Authors:** Iruka N. Okeke, Faith Wallace-Gadsden, Hannah R. Simons, Nicholas Matthews, Amy S. Labar, Jennifer Hwang, John Wain

**Affiliations:** 1 Department of Biology, Haverford College, Haverford, Pennsylvania, United States of America; 2 Wellcome Trust Sanger Institute, Genome Campus, Hinxton, Cambridge, United Kingdom; 3 Health Protection Agency, Colindale, London, United Kingdom; University of Münster, Germany

## Abstract

**Background:**

Enteroaggregative *Escherichia coli* (EAEC) are defined by their stacked-brick adherence pattern to human epithelial cells. There is no all-encompassing genetic marker for EAEC. The category is commonly implicated in diarrhea but research is hampered by perplexing heterogeneity.

**Methodology/Principal Findings:**

To identify key EAEC lineages, we applied multilocus sequence typing to 126 *E. coli* isolates from a Nigerian case-control study that showed aggregative adherence in the HEp-2 adherence assay, and 24 other EAEC strains from diverse locations. EAEC largely belonged to the A, B1 and D phylogenetic groups and only 7 (4.6%) isolates were in the B2 cluster. As many as 96 sequence types (STs) were identified but 60 (40%) of the EAEC strains belong to or are double locus variants of STs 10, 31, and 394. The remainder did not belong to predominant complexes. The most common ST complex, with predicted ancestor ST10, included 32 (21.3%) of the isolates. Significant age-related distribution suggests that weaned children in Nigeria are at risk for diarrhea from of ST10-complex EAEC. Phylogenetic group D EAEC strains, predominantly from ST31- and ST394 complexes, represented 38 (25.3%) of all isolates, include genome-sequenced strain 042, and possessed conserved chromosomal loci.

**Conclusions/Significance:**

We have developed a molecular phylogenetic framework, which demonstrates that although grouped by a shared phenotype, the category of ‘EAEC’ encompasses multiple pathogenic lineages. Principal among isolates from Nigeria were ST10-complex EAEC that were associated with diarrhea in children over one year and ECOR D strains that share horizontally acquired loci.

## Introduction

Enteroaggregative *Escherichia coli* (EAEC) is a category of diarrheagenic *E. coli* defined by a characteristic “stacked brick”, “honeycomb” or “aggregative” adherence pattern to epithelial cells [1]. In spite of their association with diarrheal illness, EAEC are commonly recovered from healthy people and their epidemiology, pathogenesis and ecology are poorly understood. The current definition, based on adherence pattern likely includes pathogenic as well as non-pathogenic strains. The inherent heterogeneity within the EAEC category, as presently defined, hampers pathogenesis research as well as the development of diagnostic tools. EAEC isolates have been implicated in acute and persistent sporadic diarrhea, and outbreaks, in both industrialized and developing countries (reviewed by [2], [3], [4]). Although recent information suggests that they are among the most common diarrheal pathogens worldwide [5], [6], [7], the true burden of disease from EAEC is unknown because many epidemiological studies, including studies that focus on diarrhea-causing *E. coli*, do not seek EAEC exhaustively or at all [6], [7], [8], [9], [10]. Moreover, genetic studies that seek to understand the evolution of pathogenic *E. coli* typically exclude, under-represent or de-emphasize EAEC [11], [12], [13].

The gold standard for EAEC identification is a HEp-2 adherence test that can only be performed in laboratories able to culture human cell lines. Attempts to identify genetic markers for EAEC have principally focused on a large, partially conserved plasmid, pAA. Many putative virulence genes map to this element, and the distribution of some of these genes has been associated with disease in epidemiological studies. However, strains that lack some or all known pAA genes are consistently recovered from people with diarrhea. Additionally, strains isolated from outbreaks, including the largest documented EAEC outbreak that affected over 2,600 Japanese school children, have commonly lacked many or all pAA putative virulence genes [14], [15]. One strain, C1096 isolated from a nursery outbreak in Serbia, harbors a large plasmid completely unrelated to the prototypical pAA plasmids [16]. Thus, even though strains bearing pAA plasmids, and specifically the *aggR* gene, have been described as ‘typical’ [17], they cannot be considered archetypical pathogenic EAEC, nor is it certain that they even represent isolates with the greatest pathogenic potential. Studies of pAA genes have uncovered important adhesins and toxins but have only emphasized both the heterogeneity of the pathotype and the limitations of the evolutionary information that can be derived from markers on a potentially mosaic mobile element [18], [19], [20], [21], [22], [23], [24].

Following on the earlier plasmid-focused studies, recent research has begun to uncover EAEC chromosomal loci of interest [25], [26], [27], [28], [29] and the completion of the genomes of EAEC strains 042, 17-2 and 101-1 will allow further identification of such factors. However, chromosomal loci are yet to be associated with disease. Furthermore, heterogeneity in host response contributes to the difficulty in interpreting the results of volunteer and epidemiological studies [30], [31], [32], making it challenging to prioritize strains for future genome sequencing, other in-depth analysis or vaccine development. Population genetic studies of EAEC strains could help address some of these questions [29].

A 1999 report described an EAEC phylogeny based on multilocus enzyme electrophoresis (MLEE) [19]. No virulence factors were associated with phylogeny, although loci examined at that time were predominantly plasmid-borne. More recently discovered loci mapped onto that phylogeny illustrate that EAEC do have lineage-specific genes [25]. However genetic relationships were only studied among 44 strains from patients with diarrhea, and the strain collection was biased towards the widely-used, plasmid-detecting probe, CVD432. A second, more recent, MLEE study of isolates from Brazil again pointed to heterogeneity and failed to identify lineage-specific genes. Interestingly, it did suggest that some lineages were more likely to harbor certain genes than others, supporting the idea that certain EAEC lineages may have epidemiological significance [24]. These data are promising but it is difficult to add strains to these phylogenies, or to overlay EAEC phylogeny with that of the genus as a whole, because they are based on the highly specialized method of MLEE.

We hypothesized that certain EAEC lineages predominate among isolates from childhood diarrhea and that hypervirulent lineages would be associated with diarrhea in a strain set from a controlled study. To test this hypothesis, we performed multilocus sequence typing (MLST) on an epidemiologically informative set of EAEC isolates obtained from Nigerian children with and without diarrhea, entering them into a curated public database to which researchers from all over the world can contribute [13]. We found that EAEC strains from Nigeria belonged to multiple sequence types (STs), but that some ST complexes were predominant. ST complexes predominant in the epidemiological set from Nigeria were also represented among well-studied EAEC strains from other parts of the world.

## Methods

### Bacterial Strains

126 EAEC strains isolated in south western Nigeria during a previous epidemiological study formed the core strain collection for this work [33]. These strains, which include 73 isolates from children with diarrhea and 53 healthy controls, represent a complete epidemiological set (excluding 5 strains that are no longer viable). They were distinguished from other *E. coli* by HEp-2 adherence, the gold standard for EAEC identification [18], [33]. Therefore, although they were drawn from one geographical location, they are not biased towards any probes, and studying the entire strain set from that epidemiological study avoids other biases associated with strain selection. Additionally, Serbian and Japanese outbreak isolates C1096 and 101-1, kindly provided by Thomas Whittam, 17 other reference strains that have been phylogenetically classified by multilocus-enzyme electrophoresis in a previous study, and five isolates from locations not represented or underrepresented in that study, were employed [19], [25]. All the strains have been previously probed for several EAEC-associated genes and verified by HEp-2 adherence assays in our laboratory. Bacteria were cultured in Luria broth (LB) or LB agar and maintained at −80°C in LB: glycerol 1∶1.

### Multi-locus sequence typing and analysis

Gene fragments from the *adk*, *fumC*, *gyrB*, *icd*, *mdh*, *purA* and *recA* were amplified as described by Wirth et al [13]. Amplified DNA was prepared for sequencing using the “ExoSAP” method (Amersham Biosciences UK Ltd). Cleaned fragments were sequenced from both ends using the di-deoxy chain terminator method [34], with V3.1 Bigdye terminator chemistry [35]. Both strands of each fragment were sequenced at least once. The resulting sequencing reactions were analyzed on 3700 or 3730 ABI sequencing machines (Applied Biosystems, USA). Allele and sequence type (ST) assignments were made at the publicly accessible *E. coli* MLST database at http://mlst.ucc.ie/mlst/dbs/Ecoli/.

Phylogenetic inferences about ancestral allelic profiles and strain interrelatedness were made using eBURST version 3 http://eburst.mlst.net/ and ClonalFrame version 1.1 http://www.xavierdidelot.xtreemhost.com/clonalframe.htm [36], [37]. Sequence type complexes were defined using eBURST as groups sharing at least six identical alleles and bootstrapping with 1000 samplings. EAEC strains in this study were mapped onto the phylogeny of the *E. coli* species as a whole using MLST data available from http://mlst.ucc.ie/mlst/dbs/Ecoli/. EAEC MLST data was further analyzed by ClonalFrame [37] to investigate relationships among different sequence type complexes. ClonalFrame is a Bayesian method of constructing evolutionary histories that takes both mutation and recombination into account. For each analysis, four independent runs of the Markov chain were employed and runs were compared by the method of Gelman and Rubin [38]. Calculated Gelman-Rubin statistics for all parameters were below 1.20, indicating satisfactory convergence between tree replicates. A 75% consensus tree was created for the EAEC isolates. Mutation and recombination rates were computed using ClonalFrame as described by Didelot and Falush [37].

### Plasmid replicon typing

Three multiplex panels comprised of 18 primer pairs were used to identify plasmid replicons in EAEC strains by PCR as described by Johnson et al [39]. Because this protocol does not amplify the FIIA replicon from EAEC strain 042, we additionally screened the strains for this replicon using primers REP042F and R (Table S1). Strain carrying the sequenced plasmids pMAR-7, pAA2 (042), pB171, pHCM1 and pED204 were used as controls [40], [41], [42], [43].

### Antimicrobial susceptibility testing

Susceptibility testing was performed in accordance with the Clinical and Laboratory Standards Institute (CLSI, formerly NCCLS) disk diffusion method [44]. Bacterial suspensions were plated onto the surface of Mueller-Hinton plates. Disks containing ampicillin (10 µg), tetracycline (30 µg), trimethoprim (5 µg), nalidixic acid (30 µg), chloramphenicol (30 µg), sulfonamide (300 µg), streptomycin (10 µg), and ciprofloxacin (5 µg) (Remel) using a disc dispenser. Plates were incubated overnight at 37°C. The diameter of inhibition zones was measured to the nearest millimeter and interpreted according to the NCCLS standards [44]. *E. coli* K-12 C600 and *E. coli* NCTC 10418 were used as controls.

### DNA hybridization

Colony blots were prepared using Whatmans 541 filter paper (Whatman, England). A 1,175 bp *Rsa*I-*Bam*HI fragment from Tn*21* and a 1,560 bp *Sph*I-*Hpa*I fragment from Tn*7* were used as *intI1* and *intI2* probes respectively [45], [46]. A 414 bp tetracycline resistance gene probe was excised from pACYC184 (New England Biolabs) using *Eco*RI and *Sca*I. A 787 bp chloramphenicol acetyl transferase gene probe was excised from pACYC184 with *Eco*RV and *Nru*I. Both fragments were purified by gel extraction and labeled by random priming with ^32^P α d-CTP employing a commercially available labeling kit (Amersham Pharmacia Biotech, NJ). Un-incorporated nucleotides were removed by passage through Sephadex G50 micro-columns (Amersham Pharmacia Biotech). Hybridization was carried out under high stringency conditions using standard techniques [47] employing a hybridization buffer composed of: 5X SSC, 0.5% sodium dodecyl sulphate, 10 mM EDTA, 1X Denhardts solution and 100 µg/ml sonicated salmon sperm DNA. Colony blots were hybridized at 65°C overnight, washed with 0.1X SSC, 0.1% SDS at 65°C and exposed to X-ray film at −80°C overnight. *E. coli* DH5α, DH5α (pACYC184) and cell-detaching *E. coli* strains that had been characterized in previous studies [48] were used as controls.

### PCR for chromosomal genes

Lysine decarboxylase genes and activity were detected as described previously [49]. Oligonucleotides used to identify virulence and antimicrobial resistance loci are listed in Table S1. They include primers designed to amplify three regions in the 042 Tn*2411*-derived resistance island as well as the *pstS* and *glmS* integenic region in *E. coli* K-12, the island's insertion site. PCR amplifications were performed using 1 unit recombinant *Taq* polymerase enzyme with PCR buffer from Invitrogen, 2 mM MgCl_2_ and 1 µM of each oligonucleotide primer in each reaction. All amplifications began with a two-minute hot start at 94°C followed by 30 cycles of denaturing at 94°C for 30 s, annealing for 30 s and extending at 72°C. PCR reactions were templated with boiled bacterial colonies. The primers, annealing temperatures, extension times and positive control strains used for each amplification are indicated in Table S1.

### Statistical analysis

Statistical tests of significance were conducted using the Chi-squared and Fisher's exact two-tailed tests on Epi-Info version 3.3 (CDC, 2004). Where applicable, a Mann-Whitney U test was performed.

## Results and Discussion

### MLST phylogeny for EAEC agrees with an earlier EAEC MLEE phylogeny

We performed MLST on 19 of the best-characterized EAEC strains. These strains cover a broad range of MLEE electrophoresis types defined by Czeczulin et al. and contain a variety of virulence gene combinations (Table S2) [19], [25], [50]. For the most part, MLEE-defined clades correspond to MLST complexes. The MLEE EAEC1 cluster, comprised of ECOR phylogenetic group A strains, is largely composed of the *E. coli* MLST ST10 complex. The MLEE EAEC2 cluster, comprised of ECOR system group D strains, belong to, or share four or more alleles with, the MLST ST31. A third cluster defined by MLEE, the AA/DA cluster, which includes diffusely-adherent *E. coli* as well as EAEC, were less closely clustered by MLST. They include strains belonging to ST complexes: 10, 23, 40, or 448 (Table S2). With one exception, all of the strains that did not cluster by MLEE belonged to other complexes or singleton STs. The 19 well-characterized strains spanned most, but expectedly not all, of the diversity seen in the collection from Nigeria. Six of the seven most common MLST clonal complexes defined in this study were represented in this test collection.

We examined the distribution of nine plasmid borne and eight chromosomal loci in the 19-strain reference collection. The distribution of most virulence genes sought among these strains did not associate with either MLST or MLEE-derived phylogeny (Table S2). The heme transport outer membrane receptor (*chuA*) and *fepC*-PAI (that is the *fepC* gene within the *prrA-modA-fepC* pathogenicity island), which were present only in ST31 and ST394 complex and related strains. However *chuA* is common to the ECOR group D lineage, to which these strains belong and *fepC*-PAI appears to show a similar distribution [25], [51]. Overall, the distribution of virulence and other genes was largely heterogeneous outside ST complexes 10, 31 and 394 later.

### EAEC plasmids belong to diverse incompatibility groups

Most loci associated with EAEC are plasmid-borne and distribution of plasmid-borne genes has previously been reported not to associate with EAEC strain phylogeny [19]. Nonetheless, given that our data reveal a few major and several minor EAEC lineages, we sought to determine whether a conserved plasmid backbone defined EAEC or whether certain EAEC lineages carried plasmids of a common origin. We subjected the 19 strains from the reference collection to plasmid replicon typing using a multiplext PCR protocol, which detects replicons from 22 plasmid incompatibility groups, described by Johnson et al [39]. Additionally, because the FIIA replicon (which is found in EAEC strain 042 and a number of enteric pathogens) is genetically diverse [52] and not detected by the Johnson et al [39] protocol, we designed primers specific for the pAA2 replicon. These primers also detect FIIA replicons from the virulence plasmids of EPEC strains B171 and E2348/69.

As shown in Table S2, the IncFIIA replicon seen in 042 (FIIA_042_), IncP, IncFIB, IncY and FIA replicons were common among the reference EAEC strains. The FIIA_042_, replicon was amplified from all but three EAEC strains. However this replicon is also commonly present in enteropathogenic, enterotoxigenic and cell-detaching *E. coli*. Twelve of the 19 reference strains were positive for more two or more of the replicons sought. No replicon was common to all the EAEC strains and none of nine other plasmid-borne genes showed absolute association with a specific replicon type. This includes the CVD432 probe locus, which is used as a marker for aggregative plasmids (pAA) that define ‘typical’ EAEC [17], [53]. A conserved plasmid backbone may not exist for pAA genes and genes associated with ‘typical’ strains, such as those encoding the antiaggregative protein (*aap*) and its secretion system (*aat*), may instead represent loci that have integrated into different plasmids. This idea is supported by the dissimilarity among three recently sequenced pAA plasmids [54]. Although these all bear a FIIA_042_, replicon (often with other replicons) and are roughly syntenic in the *aat* to fimbrial gene regions, the rest of the molecules differ in both gene content and organization [54]. Association of aggregative adherence genes with a broad range of plasmids, provides an explanation for the high prevalence of EAEC worldwide. A similar phenomenon has been used to explain the broad dissemination of antimicrobial resistance genes like *sul2* and *dfrA1* [55], [56].

### EAEC belong to multiple lineages

**Figure 1:**
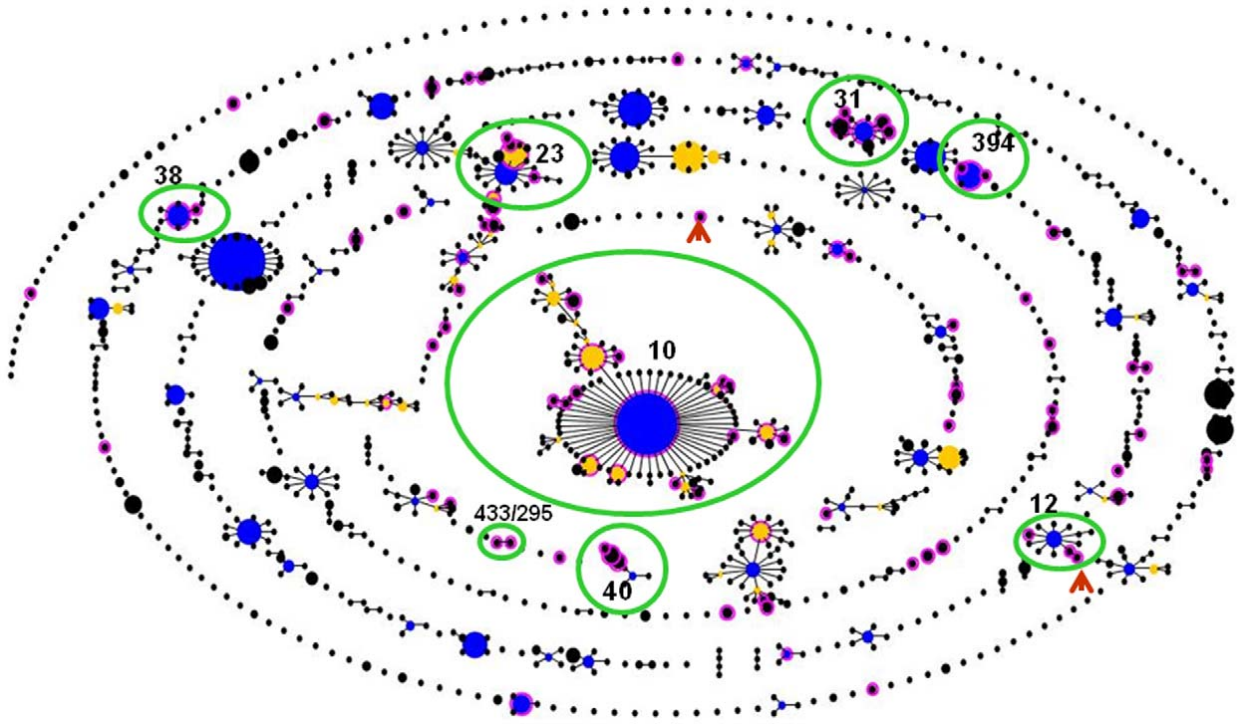
BURST output for strains in the *E. coli* MLST database with STs containing EAEC strains ringed in pink. The size of the node is proportional to the number of isolates in the STs. Blue nodes represent predicted founder STs and sub-founders are indicated in yellow. All other STs marked as black dots. STs 12, 40 and the six clonal complexes containing four or more of the EAEC strains from this study are circled in green. Brown arrows point to the two outbreak isolates.

In October 2010, there were 3344 isolates in the *E. coli* MLST database belonging to 1,985 STs and the ‘Based Upon Related Sequence Types’ (BURST) algorithm placed them in 103 clonal complexes, as defined by using a group definition of six alleles. We identified 96 of these sequence types among 150 EAEC strains in this study (Table S3). As shown in Figure 1, the EAEC strains were distributed throughout the *E. coli* phylogeny although a number of ST complexes that are abundant in the *E. coli* MLST database did not contain any EAEC strains. Common *E. coli* ST complexes that were devoid of EAEC strains included ST complexes 69, 73, 95 and 131, all of which are predominantly comprised of extraintestinal *E. coli* and belong to the B2 phylogentic group [13], [57], [58].

**Figure 2:**
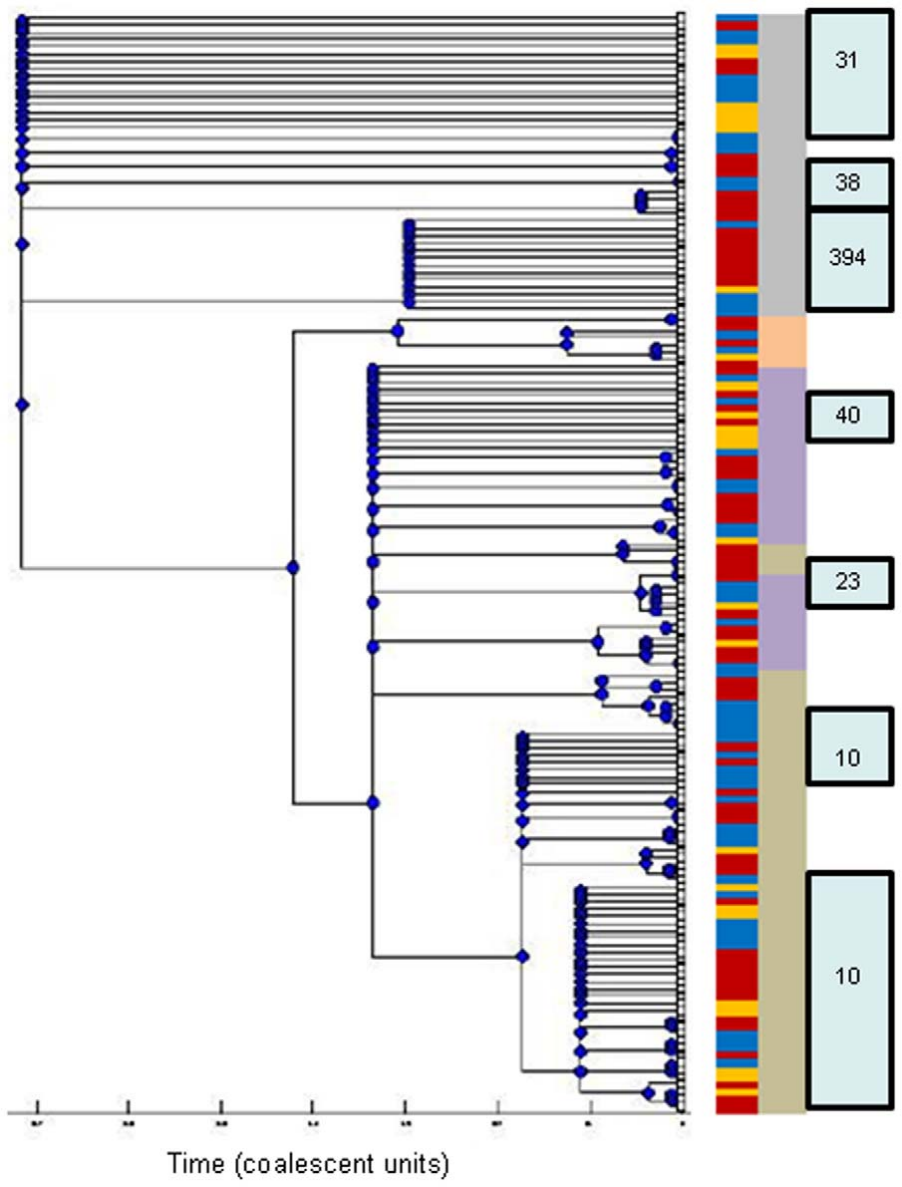
75% Consensus ClonalFrame tree for MLST data from 150 EAEC strains including strains from Nigerian children with diarrhea (Red), healthy Nigerian children (Blue), and cases of diarrhea from other parts of the world (Yellow). The tree incorporates recombination as well as mutation. The principal *E. coli* sub-clades corresponding to the four major groups originally defined by MLEE - A, B1, D and B2 - are marked to the right of the tree respectively in brown, purple, grey and orange. Boxed numbers to the right of the tree indicate strains belonging to the most common ST complexes identified by eBURST.

Ninety-six (64%) of the 150 EAEC strains from this study belonged to 16 ST complexes. Sixty (40%) of the strains fell into three main groups with predicted ancestral STs 10, 31 and 394 (Table S3). A 75% consensus tree constructed using ClonalFrame identified similar groups as eBURST, with few exceptions (Figure 2). Both BURST and ClonalFrame distinguish ST complexes 31, 38 and 394, along with four strains sharing four alleles with these complexes as a separate lineage, which corresponds to the MLEE-defined ECOR D group (Figure 2). The remainder of the EAEC strains appear much more diverse, although the ST10 complex, belonging to ECOR phylogroup A, represents a highly-prevalent delineated sub-group (Figure 2). ECOR group A strains predominate in West Africa [59], which may provide one explanation for the fact that ST10 was the most common seen in this study. Ninety (60%) isolates did not belong to predominant complexes. eBURST placed 37 of these isolates into 13 clonal complexes, each containing only 2–4 isolates and 53 of them belonged to 43 singleton STs, nine of which were identified more than once (Table S3).

The 75% consensus tree generated with ClonalFrame agreed, for the most part, with the phylogeny inferred by eBURST (Figure 2). The tree topology is similar to that originally produced for *E. coli* as a whole by MLEE, and ratified by MLST and other methods, delineating the major clades defined by the ECOR collection: A, B1, B2 and D [11], [13], [59]. Strains belonging to all four of these clades were identified among EAEC, a finding that was made in a previous study that included only 11 EAEC strains [11]. In that study, only one of the 11 isolates belonged to the B2 group and we find that in the current examination of 150 strains, the isolates were almost evenly distributed among A, B1 and D groups but only 7 (4.6%) of the strains evaluated in this study clustered in B2, a group that is largely comprised of uropathogenic, neonatal meningitic and other extra-intestinal *E. coli* pathogens (Figure 2). While this suggests that EAEC strains may not be associated with the B2 lineage, it is important to note that a few strains, including, strain 101-1, from a large outbreak in Japan, clustered with this clade. As this study focused on EAEC isolates from Nigeria, and the B2 group has previously been reported to be more common in Asia than in Africa [59], it is possible that other lineages are more prominent elsewhere.

The role of recombination, relative to point mutations, in *E. coli* evolution is uncertain, with different studies and methods producing different rate estimates [13], [60], [61]. Some studies have found that in *E. coli* and other organisms where there is no consensus on recombination rates, that recombination occurs more commonly in some lineages of a species than in others [13], [62]. Among the EAEC sequence-typed in this study, ClonalFrame identified strong evidence for recombination at all principal nodes of the tree. The relative frequency of recombination, as compared to point mutation, (ρ/θ), computed using ClonalFrame was 1.333 in the complete EAEC set (Table 1). This was greater than rates computed for the *E. coli* species by Perez-Losada et al (who estimated recombination at essentially zero [60]) but comparable to those proposed by Wirth et al (0.321–2.139 [13]) and Touchon et al (close to one [63]). However, as with Wirth et al, we found considerable variation in recombination for different lineages. Most notably, recombination rates were lower in ST10-complex EAEC and strains sharing up to four alleles with this group compared with all EAEC (Table 1), a finding that has previously been reported for the ECOR-defined group A compared to the species as a whole [13]. The net consequence of recombination, which is measured by the rate at which a given nucleotide is substituted by recombination versus point mutation (r/m), demonstrates that a change is almost twice as likely to be produced by recombination in the ST31 complex and its triple-locus variants than in ST10 and triple locus variants (Table 1).

**Table 1:** Mutation and recombination rates inferred by ClonalFrame analysis.

	All EAEC	ST31/38/394 complexes and triple-locus variants (ECOR D EAEC)	ST10 complex and triple locus variants (largely ECOR A EAEC)
Mutation rate (θ)	26.8984715(13.87313 43.33993)	2.1482595(0.730997 4.233684)	5.03401175(2.079768 9.231654)
Relative frequency of recombination, compared to point mutation in genetic diversification (ρ/θ)	1.33271125(0.653744 2.454614)	1.34384(0.384195 3.916594)	0.792226(0.234087 1.955358)
Relative impact of recombination, compared to point mutation in evolutionary history (r/m)	3.26354598(1.881123 5.470586)	4.522816(1.558328 11.61871)	2.78584325(1.034104 6.098995)

Data represent combined parameter values (from four ClonalFrame runs in each case) and 95% credibility intervals.

We examined the *E. coli* MLST database at mlst.net (http://mlst.ucc.ie/mlst/dbs/Ecoli/) for other strains present in predominant EAEC STs. As at October 2010, *E. coli* ST394 complex contained 16 isolates (12 from this study) and all but three members of this complex were annotated as EAEC or *E. coli* from diarrhea. (One isolate is annotated as unknown and two are urinary tract infection isolates). The ST394 complex may represent an EAEC-specific complex but no other ST-complexes containing more than three EAEC strains were EAEC-specific. The most common clonal complexes in which EAEC strains were identified, including those with predicted ancestors ST10, ST23, ST31 and ST38 contained strains belonging to multiple pathogenic categories as well as likely commensals and, with a few exceptions (most notably STs10, 31, 38 and 394), EAEC strains were less likely to belong to founder or sub-founder STs (Figure 1, Table S3).

MLST data for 33 EAEC strains not represented in our collection had been deposited in the *E. coli* MLST database as at October 2010. Eight of these isolates belonged to the ECOR A group ST10 complex (ST10 = 6, ST34 = 2) and five to the ECOR B1 group ST40 complex (ST40 = 2, ST200 = 3). Three isolates belonged to ST31 complex (ST31 = 2, ST130 = 1) and three others to other ECOR group D STs (ST30 = 2, ST414 = 1). All but two of these isolates were from Europe and, with the exception of ST30, all these STs were represented within our collection. A further 13 strains belonged to novel STs and each occurred only once. Six of these isolates were from Egypt, a source country unrepresented in our collection. Overall, our data and the data from other investigators suggest that EAEC strains belong to multiple lineages and that the aggregative adherence phenotype is one that is convergent. However, there are predominant and globally disseminated lineages among strains currently defined as ‘enteroaggregative’ and these include the ST complexes 10 (ECOR A), 40 (ECOR B1) and 31 (ECOR D).

### EAEC outbreak isolates

Molecular characterization of strains from epidemiological studies can bring to light overlooked outbreaks [64]. ST38 complex, and a clonal complex containing STs 435, 464 and 557, were the most prevalent EAEC clonal complexes in the Nigerian collection that were never recovered from healthy children. Three ST38 complex isolates belonged to the founder ST and the fourth was but a single-locus variant, which with the epidemiological data is suggestive of virulence potential or an outbreak. Two ST38-complex isolates were recovered from children residing in rural Akinlalu (7°28′N, 4°26′E) and the other two from adjacent Ayedade (7°28′N, 4°21′E) (Figure S1). Since the two zones are only approximately 7 kilometers apart, an outbreak or localized endemicity is possible. However, the number of ST38 complexes is small, no ST38-complex strains were recovered from elsewhere in this study and the database contains a wide range of pathotypes within ST38.

The two isolates from previously characterized EAEC outbreaks that were sequence-typed in this study did not belong to predominant EAEC STs. This is in contrast to enterohemorrhagic *E. coli* and enteropathogenic *E. coli*, for which the best documented lineages are also the predominant causes of outbreaks [65]. Strain C1096 was isolated during a neonatal outbreak in Serbia, and is unique to singleton ST490. The largest documented EAEC outbreak occurred in Japan and was caused by strain 101-1 [14], which is the single strain in ST493 (ST complex 12) (Figure 1, Table S3). We recently showed that strain 101-1 contains pathoadaptive deletions in the lysine decarboxylase genes [15], [49]. We therefore screened strains G30a (ST438) and E74 (ST476), both ST12-complex strains and single-locus variants of ST493, for lysine decarboxylase activity and the *cadABC* genes as previously described [49]. Both strains contained intact and functional lysine decarboxylase genes (Table S4).

Given that EAEC are among the most prevalent diarrheagenic pathogens, spread of some outbreak strains might actually be promoted by their dissimilarity to other EAEC, and therefore a large susceptible population. It is also possible that outbreak strains like C1096 and 101-1 have emerged locally and then dissipated because of fitness disadvantages. Our findings support the idea that minor genetic changes may result in the evolution of hypervirulent strains from lineages with low-level virulence. However evaluation of more ST12-complex EAEC strains, which were uncommon in the collection from Nigeria, is needed to test this hypothesis.

### ST10 complex, the most common EAEC lineage, is associated with diarrhea in children over one year of age

A total of 32 (21.3%) of the EAEC strains evaluated in this study clustered within a clonal complex with eBURST predicted ancestor ST10, making this the most common group among the EAEC strains profiled (Table S3). ClonalFrame predicted the same cluster with minor differences (Figure 2). ST10 is one of the most common STs in the *E. coli* MLST database (Figure 1) and, although enteropathogenic, enteroinvasive, enterohemorrhagic and meningitic *E. coli* typically do not belong to the ST10 complex, many ST10 strains do belong to other *E. coli* pathotypes. For example, although all EAEC strains used in this study were negative for heat-stable and heat-labile enterotoxins (Table S4), ST10 is the predominant enterotoxigenic *E. coli* ST [18], [33]. Nonetheless, ST10 is significantly overrepresented among EAEC, compared to all strains in the database (p<0.0001), leading us to examine this sequence type and related strains more closely.

ST10-complex EAEC strains were not significantly associated with disease in the isolates from Nigeria overall but they showed an association with diarrhea in children 10 months old or older (p<0.05, Mann-Whitney U test for the complex irrespective of whether defined by eBURST, ClonalFrame or both). Of the ten ST10 complex (eBURST) strains isolated from apparently healthy children, only one was isolated from a child older than nine months. By contrast, six of the 15 isolates from children with diarrhea were recovered from children aged 10 months or older (p<0.02, Fisher's exact test).

All but one of the ST10 complex strains in our EAEC collection, and all of the Nigerian isolates, hybridized with at least one pAA-derived probe (Table S4). All ST10 and related isolates were negative for *chuA*, which is seen in the ECOR B2 and D lineages. No other chromosomal loci sought in this study were identified in more than two ST10-complex strains. All ST10 complex isolates from Nigeria were resistant to at least two antimicrobial classes. ST10 complex-EAEC resistance patterns were highly variable and there were no predominant resistance profiles, in contrast to ST complexes 31 and 394 described below (Table S4). Ten multiply-resistant ST10 complex strains were able to transfer one or more resistance markers via conjugation [18] suggesting a high prevalence of mobile elements within this ST complex.

Early prototypical EAEC strain 17-2 from Chile, which produced diarrhea in only one of 24 adults in one study and none in a second study, belongs to ST10 [31], [32]. As a result of poor diarrheagenicity in North American adults, interest in this strain has waned. However, as Nataro [66] has observed, while outbreak information and volunteer studies can authoritatively implicate a putative pathogen in disease they cannot prove that a given strain is non-pathogenic. Our data suggest that immune protection to this lineage may be acquired early in life, given the high prevalence of ST10 in general, so that virulence of 17-2 and related strains cannot be assessed in adult volunteers. This is supported by the fact that in one study 13 of 19 volunteers post-screened in another and 40 of 60 volunteer candidates pre-screened had antibodies to a 14 KDa 17-2 protein before they were infected with the strain [32].

In contrast to adult human models, we found that two ST10 complex strains 17-2 (ST10) and 60A (ST34) were among the most virulent in our *Caenorhabditis elegans* model for EAEC [49]. Fernandez-Prada et al [67] reported that, like *Shigella*, ST10 EAEC strain 17-2 caused cytolysis of human monocyte-derived cell line and apoptosis in the murine cell line JM774 which they attributed the cytotoxicity to hemolysin. In addition, the ST10 complex EAEC strains show exceptional biofilm forming capacity compared to other EAEC strains [50], [68]. The pathogenic potential shown in these models, the high prevalence of ST10-complex strains in this study – from Nigeria and elsewhere - together with the association of ST10 complex EAEC with disease among weaned Nigerian children, suggest that pathogenesis research on these strains should be resumed and that future studies evaluating the pathogenic role of ST10 complex EAEC need to be suitably age stratified. ST10 *E. coli* appear to become enterotoxigenic by the acquisition of plasmid-borne virulence factors alone [69]. It is possible that the same is true for aggregative adherence in this lineage. In a recent report, ST10 complex isolates were shown to commonly carry horizontally acquired genes encoding extended spectrum β-lactamases [70]. This possible propensity to acquire mobile elements may not be unrelated to the presence of error-prone repair genes on *aggA*-bearing aggregative plasmids, which are harbored by many ST10 EAEC isolates [50].

### An EAEC lineage with a conserved antimicrobial resistance island

**Figure 3:**
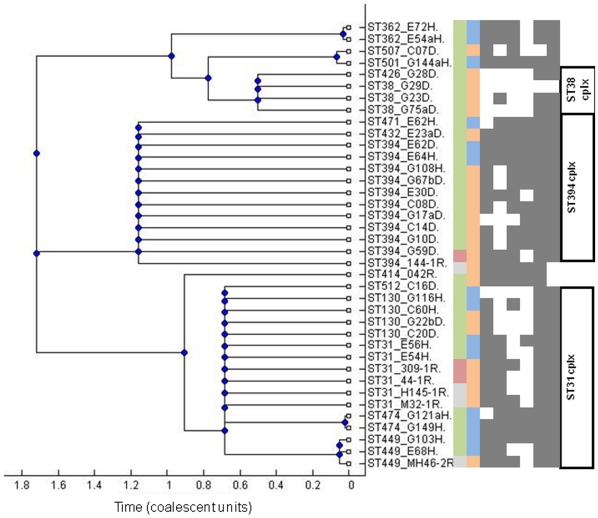
Presence of absence of markers of the 042 antimicrobial resistance island and other chromosomal loci in EAEC belonging to phylogenetic group ECOR D mapped onto a 75% consensus tree. Columns from left to right of the matrix indicate strain source (green  =  Nigeria, red =  Thailand, grey = Peru), host status (orange = diarrhea, blue = healthy), columns 3–8 *lpf* A, *cat* 3, *insA (Tn 1723), hra1, chuA, iucA* (grey  =  positive, white  =  negative).

The most distinct EAEC lineage identified by eBURST and ClonalFrame in this study corresponds to the MLEE-defined ECOR D clade (Figure 2). The cluster includes prototypical and genome-sequenced EAEC strain 042 (ST414) from Peru [29]. As shown in Figure 3, this cluster is comprised largely of ST31-, ST38- and ST394 complexes. While all but one ST394 EAEC were identified in the Nigerian collection, ST31 complex strains from Peru and Thailand were identified in addition to strains from Nigeria. From the Nigerian collection, ST394 complex strains were predominantly recovered from children with diarrhea whilst ST31 complex strains were predominantly recovered from healthy children. ST394 (n = 10) was the most common EAEC ST after ST10 (n = 13).

Strains belonging to ST31, ST38 and ST394 complexes share between two and five alleles with ECOR D ST69, which includes uropathogenic *E. coli* strains belonging to the internationally disseminated multiply-resistant clonal group A [57]. We have previously reported that ST31- and ST394-complex EAEC strains are multiply-resistant EAEC, pAA-positive, *chuA*-positive, and almost always bear a flagellin gene encoding the H18 antigen [71]. Seeking to determine whether these strains share other chromosomal loci outside the core *E. coli* genome, we screened them for *hra1*, a chromosomal gene encoding an accessory colonization factor, which we have recently described in ST31-complex EAEC strain 042 [72]. Seventeen (48.6%) of EAEC strains in the ECOR D group but only 25 (13.0%) of other EAEC strains possessed this gene (Table S4).

**Figure 4:**
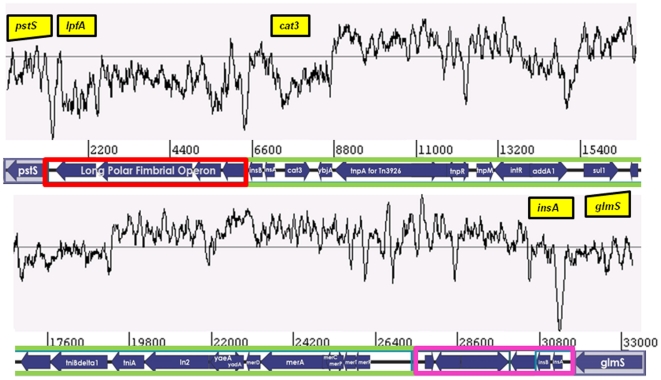
Gene order and arrangement in the 042 antimicrobial resistance island inserted between *pstS* and *glmS* on the *E. coli* genomic backbone (shaded). GC content is plotted on top of the gene-content schematic and regions used to screen for the different segments of the island are indicated by yellow boxes. The island consists of a 5′ long-polar fimbrial operon (outlined in red) with lower overall GC content, and a resistance region similar to that of Tn21 (outlined in green). The 3′ end of the island is identical to part of Tn*1723*. The resistance region and the 3′ end comprise Tn*2411.*

Most ST31 and ST394 complex EAEC hybridized with probes for antimicrobial resistance genes *cat* and *tet* and class 1 integrase gene *intI1*, loci that were occasionally, but not commonly present in other EAEC (Figure 3, Table S4). In the recently published genome of EAEC strain 042, the *intI1* gene lies within a mosaic chromosomal island of plasmid origin, which is composed of a 4,988 bp long polar fimbrial operon and Tn*2411*, a 20,321 bp Tn*21*-like element bearing a class 1 integron with an *aadA* cassette encoding resistance to streptomycin. Tn*2411* also carries chloramphenicol, tetracycline and mercury resistance genes [29]. This antimicrobial resistance island is inserted between the *pstS* and *glmS* open reading frames on the K-12 backbone (Figure 4).

To determine the prevalence of this island in ECOR D EAEC strains we first used primers spanning the *pstS-glmS* junction. These primers, pstSF and glmSR, failed to generate a predicted 945 bp amplicon for all the ST31 and ST394 complex, and related, isolates, but not ST38 complex strains. Primers specific for four genes on the island, *lpfA*, *cat3*, *intI1* and *insA*, were used to test the content and distribution of the island in EAEC strains by PCR. As shown in Figure 3, the island is a mosaic and only five strains possess all four island loci.

Eight ST31 and ST394 complex strains and 13 EAEC strains from other STs, were positive for only one island marker, *lpfA* (Table S4). This suggests that these isolates might carry a smaller version of the island, which is present in EHEC O157:H7 strains, and if so that the fimbrial island may have been acquired ahead of the resistance region in a strain ancestral to ST31 and ST394. Fifteen EAEC strains that did not have the resistance island or the *lpfA* gene inserted at this site but still had a *pstS-glmS* insertion were found to carry Tn*7*, a transposon known to preferentially integrate at this site. Tn*7* carries a class 2 integron with *dfrA1*, *sat* and *aadA* and genes encoding resistance to trimethoprim and the aminoglycosides [73]. Seven of the Tn*7*-positive strains belonged to ST10 complex and none of them were positive for any of the ST31/ST394 resistance island-markers.

The mutual exclusivity of these two resistance islands is interesting and the presence of at least one of them in a substantial proportion of EAEC strains screened explains several anecdotal reports associating antimicrobial resistance with EAEC [18], [48], [74], [75], [76], [77]. It is possible that antimicrobial use over the last half-century has provided selective pressure that permitted expansion of resistant EAEC in a manner similar to what has occurred in prominent uropathogenic lineages. For example, ‘Clonal Group A’ strains (ST69) contain a 23 Kb resistance island inserted at *leuX*, which contains *cat*, *dfrA7*, *aadA*, *sulI* and *bla*_TEM_ conferring resistance to chloramphenicol, trimethoprim, streptomycin, sulphonamides and ampicillin respectively [78]. Resistance islands could be an important feature ensuring success of pathogenic *E. coli* lineages in this era of antimicrobial overuse.

Recent studies have shown that genomic islands integrated in the chromosomes of *Vibrio cholerae*, *Yersinia*, *Salmonella*, and *Shigella* isolates can be mobilized [79]. We tested the mobility of the ST31 resistance island. Solid and liquid mating experiments were performed using twelve ST31 and ST394 strains as candidate donors and a nalidixic acid resistant derivative of *E. coli* K-12 C600 as recipient. Matings were performed at room temperature or 37°C for 3 hours or overnight but no transconjugants carrying island genes were isolated. LexA autoproteolysis following DNA damage has been shown to induce transfer of some genomic islands but we were unable to induce transfer by UV induction. We therefore conclude that the island is not mobile under the conditions tested and at our level of detection (about 1 in 10^9^).

Computing the ratio between the lengths of tree external and internal branches provides some information about the demographics of bacterial populations. For the complete EAEC data set, ClonalFrame-inferred ratios of external-to-internal branches were significantly lower than the coalescent expectation (p = 0.00905, 0.0181) (Figure S2). This usually is indicative of a population that subdivided or split, but allows for occasional recombination but this interpretation must be made with caution in *E. coli* where the lengths of recombined sequence are often short and can overlap, thereby potentially producing an exaggerated influence on branch lengths [37], [63], [80], [81]. Significantly lower actual ratios were seen in most other parts of the tree, to varying degrees. However, the internal-external branch ratio for the ECOR D lineage (p = 0.7098), in contrast to the ECOR A lineage, including ST10 complex (p = 0.04405), is not significantly different from the coalescent model prediction suggesting clonal expansion of this lineage (Figure S2).

By definition, a pathotype consists of a sub-specific category that produces a specific disease syndrome in a susceptible host by virtue of common virulence factors and mechanisms. A pathogenic clone is comprised of organisms of the same pathotype with common ancestry [82]. O:H serotyping, multilocus enzyme electrophoresis and, most recently, multilocus sequence typing have assisted in defining pathotypes of extraintestinal *E. coli* and some diarrheal pathogens like enterohemorrhagic *E. coli* and enteropathogenic *E. coli*. Strains from these pathotypes carry plasmid-borne virulence genes but are defined by genomic islands acquired step-wise by pathogenic clones [57], [83]. By contrast, principal virulence factors of enterotoxigenic *E. coli* are typically plasmid-borne so that the pathotype is defined by those factors, rather than by any distinct lineage [69]. However, probably because chromosomal background is a determinant in the horizontal acquisition of mobile elements, some lineages are more likely to harbor toxigenic plasmids than others [11], [84]

Unlike the ST38-complex strains, ST31- and ST394-complex isolates were from children residing at various locations across the study area and abroad (Figure S1). Altogether, seven of the 26 isolates not from Nigeria belonged to ST31- (6 strains) or ST394 (1 strain) complex and therefore a localized outbreak cannot explain the predominance of this group. Instead, ECOR D EAEC appear to comprise a disseminated EAEC pathogenic clone, or at the very least a pathotype. Like other pathogenic clones, these strains share chromosomal and plasmid virulence loci and have a demonstrated clonal origin. ST38 complex strains are part of this lineage but do not carry the 042 resistance island. However, the presence of the island in co-clustering singleton STs 362, 501 and 507 strains strongly suggests that the ST38 complex strains identified in this study may have acquired and then lost the island (Figure 2).

### Conclusion

In this study, we developed a molecular phylogenetic framework for EAEC, which can be built upon without highly specialized methodology. The considerable diversity we observed within a single epidemiological strain set, confirms that EAEC are indeed very heterogeneous and demonstrates that the EAEC pathotype, as presently defined, is comprised of *E. coli* strains whose defining superior colonization phenotype does not reflect a common ancestry or even a manageable number of clonal groups. These findings are supported by a recent comparative study of draft genomes from EAEC strains 042 and 101-1 that identified only three ‘EAEC-specific’ genes shared between the two strains, but absent from other *E. coli* [15].

The *E. coli* genome is extraordinarily plastic, allowing for different phenotypes to emerge convergently multiple times [13], [63]. In this study, we have identified multiple EAEC subgroups of interest from children with diarrhea and healthy controls in Nigeria. The principal lineages were represented in a reference collection drawn from more diverse locations. Our data provide convincing evidence that aggregative adherence is a bacterial outcome of co-evolution of human hosts and has been acquired by different *E. coli* lineages through multiple paths. This study confirms previous reports pointing to extreme heterogeneity in the fecal *E. coli* category described as enteroaggregative and strongly suggests that ‘EAEC’ is likely a conglomerate of convergently evolved enterovirulent lineages some of which may share similar but non-identical mobile elements. Among them are the predominant ECOR group D EAEC pathotype and the ST10-complex lineage both of which this study suggests deserve further study. However we also demonstrate that strains arising from less common lineages may have important pathogenic potential and that there is probably geographical variation in the epidemiology of EAEC lineages. Although this study was largely focused on isolates obtained from a single time and place, it recovered strains that spanned the known range of *E. coli* phylogeny. Therefore no single strain can be considered representative of EAEC. Instead, there is need to identify specific virulence genes and mechanisms within lineages in order to produce a nuanced assessment of the pathogenesis and epidemiology of strains presently categorized as EAEC. Additionally, MLST studies on EAEC from controlled studies performed in other geographic locations could identify other pathogenic lineages.

## Supporting Information

Table S1 Oligonucleotide primers used in this study.
(0.06 MB DOC)

Table S2 MLST allele profiles of EAEC strains that have previously been phylogenetically categorized by MLEE by Czeczulin et al (1999).
(0.12 MB DOC)

Table S3 BURST results for 150 EAEC isolates.
(0.04 MB XLS)

Table S4 Allele profiles, virulence gene and antimicrobial resistance properties of EAEC isolates.
(0.15 MB XLS)

Figure S1 Distribution of the ratios of external branch length to internal branch length of trees resulting from ClonalFrame analysis of (A) all EAEC isolates, (B) ST10 and related strains and (C) ST31 and related strains. External/internal branch length ration (x-axis) is plotted against tree sample frequency (y-axis). The external to internal branch length ratio is significantly lower than that for trees simulated under the coalescent model for the complete EAEC data set (p<0.01) and ST10 complex (p<0.05).
(0.23 MB TIF)

Figure S2 Geographic source of ST31, ST38 and ST394 EAEC isolates. The location of central towns and villages in the zones from which Nigerian patients and controls were drawn are indicated by red markers. S, or sub-urban, indicates small towns, R, rural villages.
(0.55 MB TIF)
