# Supplementary material for: Multi-Locus Sequence Typing of Enteroaggregative Escherichia coli Isolates from Nigerian Children Uncovers Multiple Lineages
Source: PLoS One. 2010 Nov 23;5(11):e14093. doi: 10.1371/journal.pone.0014093 (PMC2990770; doi:10.1371/journal.pone.0014093)
Supplement: Table S1 — Oligonucleotide primers used in this study. (0.06 MB DOC) [file pone.0014093.s001.doc]

Supplemental Table 1: Oligonucleotide primers used in this study

| **Target gene** | **Primer** | **Primer Sequence** | **Amplicon length** | **Purpose** | **Reference** |
| --- | --- | --- | --- | --- | --- |
| *pstS*-*glmS* | *pstSF* | CAA CGG TAT TAG CGA TAA TC | 945 bp | *pstS-glmS* intergenic region | [1] |
|  | *glmSR* | TCT CTG ACA AAC ATC ACG C |  |  |  |
| *IS (Tn 1723)* | fwg2F | CTC ATC CAG TAG TC | 119 bp | EAEC resistance island | This study |
|  | fwg2R | CTC GCA AAT CGG CGA AAA |  |  |  |
| *cat-3* | hrs3F | ACC GTT GAT ATA TCC CAA TG | 614 bp | EAEC resistance island | This study |
|  | hrs3R | CTC ATC GCA GTA CTG TTG TAA |  |  |  |
| *lpf* | fwg6F | GCT ACG ATT ATG ACT AAA CC | 523 bp | EAEC resistance island | This study |
|  | fwg6R | TGA TGA CCT ACA TGA TGG AA |  |  |  |
| *aaiC* | aaiCF | TATATATCTAGAAACCTTAAATGATGAGT | 300 bp | *aaiC* from 042 *pheU* island | [2,3] |
|  | aaiCR | AAAAAATCTAGATGTCAAAATAGAGATAGGTG |  |  |  |
| *hra1* | aggl1F | ATTGCGGTTTCAGCGCTTGC | 690 bp | Hra1 accessory colonization factor | [4] |
|  | aggl1R | AAG CAG ACT TGA |  |  |  |
| repA  (042) | REP042F | ACGTGACAGAATCATGCGCT | 819 bp | FIIA replicon from EAEC strain 042 | This study |
|  | REP042R | GTGACTGATCTTCAACAAACGTA |  |  |  |
| Tn*7* | Hep74 | CGG GAT CCC GGA CGG CAT GCA CGA TTT GTA | 2.2 Kb | Resistance cassette-containing region of Tn*7* | [5] |
|  | Hep51 | GAT GCC ATC GCA AGT ACG AG |  |  |  |

**References**

1. Hwang J, Mattei LM, VanArendonk LG, Meneely PM, Okeke IN (2010) A pathoadaptive deletion in an enteroaggregative *Escherichia coli* outbreak strain enhances virulence in a *Caenorhabditis elegans* model. Infect Immun 78: 4068-4076.

2. Opintan JA, Bishar RA, Newman MJ, Okeke IN (2010) Carriage of diarrhoeagenic *Escherichia coli* by older children and adults in Accra, Ghana. Trans R Soc Trop Med Hyg 104: 504–506.

3. Dudley EG, Thomson NR, Parkhill J, Morin NP, Nataro JP (2006) Proteomic and microarray characterization of the AggR regulon identifies a pheU pathogenicity island in enteroaggregative Escherichia coli. Mol Microbiol 61: 1267-1282.

4. Bhargava S, Johnson BB, Hwang J, Harris TA, George AS, et al. (2009) The heat resistant agglutinin 1 is an accessory enteroaggregative Escherichia coli colonization factor. J Bacteriol 191: 4934-4942.

5. White PA, McIver CJ, Rawlinson WD (2001) Integrons and gene cassettes in the enterobacteriaceae. Antimicrob Agents Chemother 45: 2658-2661.
