# Supplementary material for: Multi-Locus Sequence Typing of Enteroaggregative Escherichia coli Isolates from Nigerian Children Uncovers Multiple Lineages
Source: PLoS One. 2010 Nov 23;5(11):e14093. doi: 10.1371/journal.pone.0014093 (PMC2990770; doi:10.1371/journal.pone.0014093)
Supplement: Table S2 — MLST allele profiles of EAEC strains that have previously been phylogenetically categorized by MLEE by Czeczulin et al (1999). (0.12 MB DOC) [file pone.0014093.s002.doc]

Supplemental Table 2: MLST allele profiles of EAEC strains that have previously been phylogenetically categorized by MLEE by Czeczulin et al [10]

| Strain | Location | Serotype | MLST | | | | | | | | | MLEE | | | Plasmid replicon(s) detected | *Plasmid-borne loci [10,16,38]* | | | | | | | | | *Chromosomal loci[10,16,38]* | | | | | | | |
| --- | --- | --- | --- | --- | --- | --- | --- | --- | --- | --- | --- | --- | --- | --- | --- | --- | --- | --- | --- | --- | --- | --- | --- | --- | --- | --- | --- | --- | --- | --- | --- | --- |
|  |  |  | adk | fumC | gyrB | icd | mdh | purA | recA | ST | ST complex | Ancestral group | Cluster | Electrophoresis type |  | CVD432 | *shf* | *aggA* (AAF/I) | *pet* | *aafA* (AAF/II) | *aggR* | *aap* | *impB* | *iucA* | *hra1* | *aaiC* | *fepC* (PAI) | *chuA* | *irp2* | *fyuA* | *pic* | *Ecs 2799* |
| 60A | Mexico | ND | 10 | 11 | 4 | 1 | 8 | 8 | 2 | 34 | 10 | A | EAEC1 | 1 | FIIA_042_ | + | - | + | - | - | + | + | + | + | - | + | - | - | + | + | + | + |
| H191-1 | Peru | ND | 10 | 11 | 4 | 8 | 8 | 8 | 2 | 10 | 10 | A | EAEC1 | 3 | - | - | - | - | - | - | - | - | - | + | - | - | - | - | - | - | + | - |
| H232-1 | Peru | ND | 10 | 11 | 4 | 1 | 8 | 8 | 2 | 34 | 10 | A | EAEC1 | 6 | FIIA_042_, FIA, P | + | + | - | - | - | + | + | + | + | - | + | - | - | + | + | + | - |
| 6-1 | Thailand | OR:H2 | 111 | 11 | 4 | 8 | 8 | 8 | 2 | 559 | 10 | A | EAEC1 | 9 | FIIA_042_, FIB, P | + | + | - | - | - | + | + | + | + | - | + | - | - | + | + | - | - |
| 17-2 | Chile | O3:H2 | 10 | 11 | 4 | 8 | 8 | 8 | 2 | 10 | 10 | A | EAEC1 | 9 | FIIA_042_, FIB, Y | + | + | + | - | - | + | + | + | + | - | + | - | - | - | - | - | - |
| 101-1* | Japan* | O?:H10 | 40 | 13 | 9 | 13 | 16 | 10 | 9 | 493 | 12 | B2 | INT | 11 | FIIA_042_ | - | + | - | - | + | - | + | - |  | - | - | - | - | - | - | - | + |
| 501-1 | Thailand | OR:H53 | 10 | 23 | 109 | 8 | 8 | 88 | 2 | 518 | 10 |  | INT | 20 | - | - | - | - | - | - | - | - | - | - | - | - | - | - | - | - | - |  |
| H223-1 | Peru | ND | 56 | 121 | 1 | 1 | 8 | 8 | 20 | 451 | None |  | INT | 21 | - | + | + | - | - | - | + | + | - | + | - | + | - | - | + | + | - | + |
| C1096* | Serbia* | O4:H? | 56 | 130 | 4 | 10 | 7 | 8 | 6 | 490 | 10 |  | INT | 34 | FIIA_042_, A/C | - | - | - | - | - | - | - | - | - | - | - | - | - | + | - | - | + |
| 042 | Peru | O44:H18 | 18 | 22 | 20 | 23 | 5 | 15 | 4 | 414 | 31 |  | EAEC2 | 38 | FIIA_042_, P | + | + | - | + | + | + | + | +- | - | + | + | + | + | + | + | + | - |
| 44-1 | Thailand | 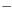O36:H18 | 18 | 22 | 17 | 6 | 5 | 5 | 4 | 31 | 31 | D | EAEC2 | 42 | FIIA_042_, FIA | + | - | - | - | - | + | + | + | + | - | + | + | + | + | + | - | - |
| 144-1 | Thailand | O77:NM | 21 | 35 | 61 | 52 | 5 | 5 | 4 | 394 | 394 | D | EAEC2 | 40 | FIIA_042_, P, Y | + | + | - | - | - | + | + | + | + | + | + | + | + | + | + | - | - |
| H145-1 | Peru | ND | 18 | 22 | 17 | 6 | 5 | 5 | 4 | 31 | 31 | D | EAEC2 | 43 | FIIA_042_, FIA | + | - | + | - | - | + | + | + | + | - | + | + | + | + | + | - | - |
| 309-1 | Thailand | O130:H27 | 18 | 22 | 17 | 6 | 5 | 5 | 4 | 31 | 31 | D | EAEC2 | 43 | FIIA_042_, FIA, P | + | - | + | - | - | + | + | + | - | - | + | + | + | + | + | + | - |
| 103-1 | Thailand | O148:H28 | 6 | 6 | 5 | 16 | 11 | 8 | 7 | 448 | 10 |  | AA/DA | 44 | FIIA_042_, Y | + | - | - | - | - | - | + | - | + | - | + | - | - | + | + | - | - |
| 435-1 | Thailand | 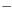 O33:H16 | 6 | 4 | 12 | 1 | 9 | 2 | 7 | 295 | 10 |  | AA/DA | 50 | FIIA_042_, FIB | + | - | - | + | + | + | + | + | + | + | + | - | - | + | + | + | + |
| 199-1 | Thailand | OR:H1 | 6 | 4 | 5 | 26 | 7 | 8 | 14 | 200 | 40 | B1 | AA/DA | 52 | FIIA_042_ | + | + | - | + | + | + | + | - | - | - | + | - | - | + | - | + | - |
| 278-1 | Thailand | O125ac:H21 | 6 | 4 | 5 | 26 | 20 | 8 | 14 | 40 | 40 | B1 | AA/DA | 54 | FIIA_042_, Y | + | - | - | - | - | + | + | + | + | - | + | - | - | + | + | + | - |
| H194-2 | Peru | ND | 56 | 4 | 12 | 1 | 9 | 2 | 7 | 433 | 10 |  | AA/DA | 53 | FIIA_042_, Y | + | - | + | - | - | + | + | - | + | - | + | - | - | + | + | + | - |

*Outbreak isolate
