# Supplementary figures and images for: Multi-Locus Sequence Typing of Enteroaggregative Escherichia coli Isolates from Nigerian Children Uncovers Multiple Lineages
Source: PLoS One. 2010 Nov 23;5(11):e14093. doi: 10.1371/journal.pone.0014093 (PMC2990770; doi:10.1371/journal.pone.0014093)

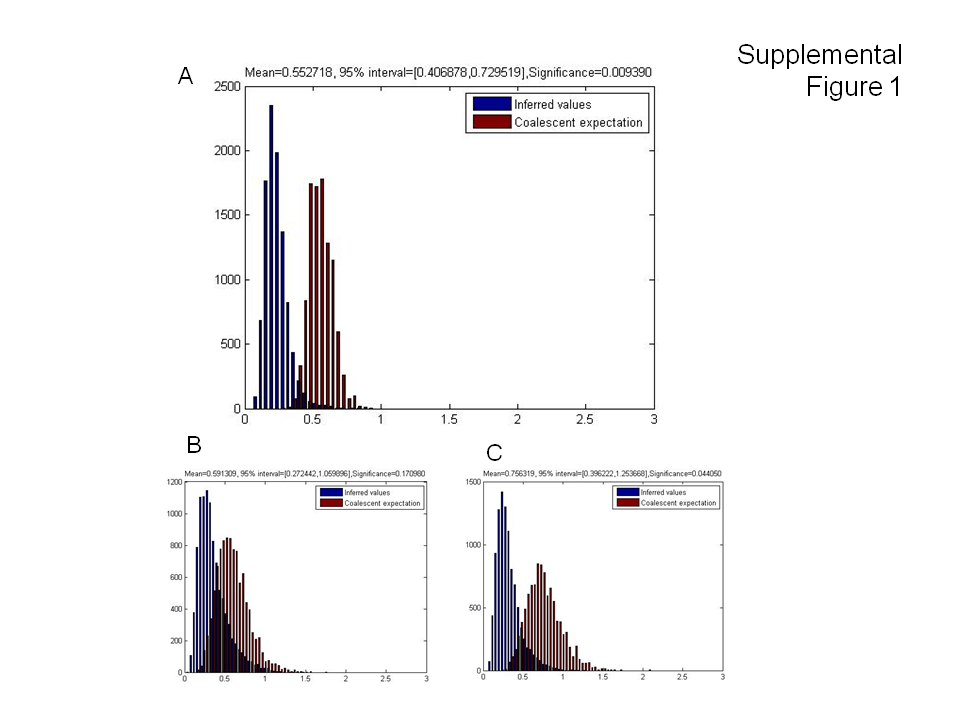

Supplement: Figure S1 — Distribution of the ratios of external branch length to internal branch length of trees resulting from ClonalFrame analysis of (A) all EAEC isolates, (B) ST10 and related strains and (C) ST31 and related strains. External/internal branch length ration (x-axis) is plotted against tree sample frequency (y-axis). The external to internal branch length ratio is significantly lower than that for trees simulated under the coalescent model for the complete EAEC data set (p<0.01) and ST10 complex (p<0.05). (0.23 MB TIF) [file pone.0014093.s005.tif]

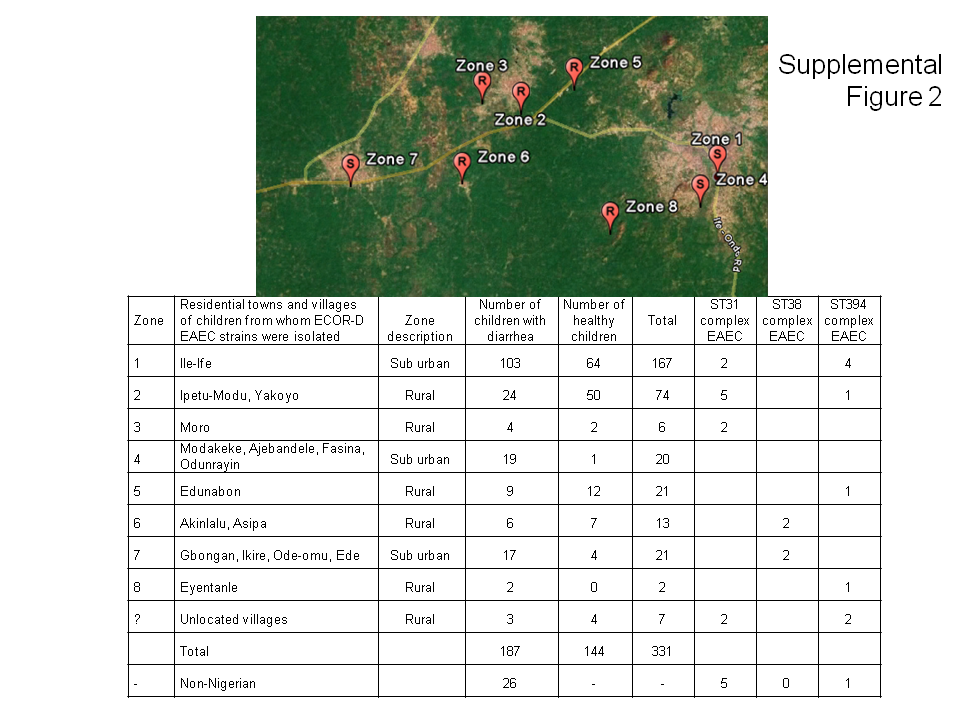

Supplement: Figure S2 — Geographic source of ST31, ST38 and ST394 EAEC isolates. The location of central towns and villages in the zones from which Nigerian patients and controls were drawn are indicated by red markers. S, or sub-urban, indicates small towns, R, rural villages. (0.55 MB TIF) [file pone.0014093.s006.tif]
